# Parenting with PTSD: A Review of Research on the Influence of PTSD on Parent-Child Functioning in Military and Veteran Families

**DOI:** 10.3389/fpsyg.2017.01101

**Published:** 2017-06-30

**Authors:** Suzannah K. Creech, Gabriela Misca

**Affiliations:** ^1^Central Texas Veterans Health Care System – Veterans Health Administration, VISN 17 Center of Excellence for Research on Returning War Veterans, WacoTX, United States; ^2^Department of Psychiatry, Dell Medical School of the University of Texas at Austin, AustinTX, United States; ^3^Institute of Health and Society, University of WorcesterWorcester, United Kingdom

**Keywords:** parenting, PTSD, child-parent functioning, parenting behaviors, child outcomes, military and veteran parents

## Abstract

Posttraumatic stress disorder (PTSD) is strongly associated with exposure to war related trauma in military and veteran populations. In growing recognition that PTSD may influence and be influenced by social support and family systems, research has begun to explore the effects that war related trauma and the ensuing PTSD may have on varied aspects of close relationship and family functioning. Far less research, however, has examined the influence of war-related PTSD on parent-child functioning in this population. This paper provides a timely review of emergent literature to examine the impacts that PTSD may have on parenting behaviors and children’s outcomes with a focus on studies of military and veterans of international conflicts since post-9/11. The review sheds light on the pathways through which PTSD may impact parent-child relationships, and proposes the cognitive-behavioral interpersonal theory of PTSD as a theoretical formulation and extends this to parenting/children. The review identifies the strengths and limitations in the extant research and proposes directions for future research and methodological practice to better capture the complex interplay of PTSD and parenting in military and veteran families.

## Introduction

The prevalence of posttraumatic stress disorder (PTSD) in samples of United States military service members and veterans who deployed to the wars in Iraq and Afghanistan has recently been estimated to be as high as 23% (Fulton et al., 2015). In growing recognition that PTSD may influence and be influenced by social support and family systems, research has begun to explore the effects that military-related PTSD may have on varied aspects of close relationship and family functioning (Vogt et al., 2016; Wagner et al., 2016). Understanding these associations is essential, as social and relationship functioning may have important implications for treatment and prevention of PTSD.

In the United States alone, estimates suggest 43% of current service members are parents to at least one dependent child (Department of Defense, 2014). Given the high rates of PTSD in those who deployed, these numbers highlight the need for improved understanding of parent-child functioning in military populations. A robust body of research has explored the effects of deployment on child adjustment (see Creech et al., 2014; Alfano et al., 2016 for reviews). Until recently, far less research had examined the influence of parental PTSD on parent-child functioning. Recent growth of studies in this area underscored the need for a new review of the literature.

## Sample of Studies and Analysis

This review examined post-9/11 and cross-cultural research on the influence of military-related PTSD and common comorbidities on parenting, child outcomes, and parent-child functioning. We also extend the cognitive-behavioral interpersonal theory of PTSD (Monson et al., 2012) to parent-child functioning. Literature searches of PsycINFO, MEDLINE, PubMed, and Web of Science from 2001 to 2016 were performed. Key words included: ‘military/soldier^∗^/arm^∗^/combat/veteran^∗^’ AND ‘Iraq/Afgha-nistan/OIF/OEF’ AND ‘parent^∗^/maternal/paternal’ AND ‘PTSD/posttraumatic stress’ AND (‘child^∗^’ AND ‘outcome/problem/disorder’). Hand-searches were conducted via the reference lists of selected papers. A total of 83 studies were retrieved and assessed according to the following inclusion criteria: (1) reported data on parenting and/or child outcomes of military personnel or veterans deployed to Iraq and/or Afghanistan, (2) included measures of military parent’s PTSD; (3) peer-reviewed and reported in English. A quality appraisal of the studies (on criteria including population, representativeness, sample size, validity of outcome measures and statistical power of results) was undertaken independently by the two authors and inclusion was determined by agreement. This systematic search identified 20 studies (summarized in **Table 1**). Although the aim was to identify research globally, all studies that satisfied the inclusion criteria were exclusively United States based. Given the heterogeneity of studies in terms of samples, design and outcome measures used (which precluded the use of meta-analysis) a narrative synthesis was employed as method of analysis.

**Table 1:** Summary of studies included in the review^1^.

Study (authors, year)	Sample/Respondents	Study design	Measures	Main findings
(1) Allen et al., 2010	434 couples consisting of active duty Army husbands married to civilian wives.	Cross-sectional	PCL, PAI	PTSD symptoms associated with decreased parenting alliance.
(2) Blow et al., 2013	1,143 National guard couples/parents	Cross-sectional	AUDIT, PSS, PCL, BDI-2	Depression, alcohol use, PTSD symptoms were significantly correlated with parenting stress for service members and spouses.
(3) Brockman et al., 2016	184 male National Guard or Reserve military service members, partners and target child between 4 and 13 years of age	A subset of military service members and their families participating in a larger intervention study	DRRI-2; PCL-M;AAQ-II; videotaped interactions	Service members experiential avoidance and PTSD symptoms associated with increased distress avoidance during the observed interaction with children; experiential avoidance associated with less positive engagement with children.
(4) Creech et al., 2016	134 women veterans	Cross-sectional	DRRI-2; PCL;AUDIT; CSI; PSOC	No significant association between PTSD symptoms or alcohol misuse with parenting confidence or parenting satisfaction; post-deployment stress predicted parenting satisfaction.
(5) Davis et al., 2015	282 National Guard/Reserve fathers	Baseline data from longitudinal prevention study	PCL; DAS; Fathers’ parenting practices – observation	PTSD symptoms, negative life events, and battle experiences not associated with observed parenting.
(6) Gewirtz et al., 2010	468 National Guard fathers from a brigade combat team	Prospective 1-year longitudinal study	PCL, APQ, parent-child relationship quality	Increases in PTSD symptoms over time associated with self-reported poorer parenting practices. PTSD symptoms predicted parenting challenges independent of their impact on couple adjustment.
(7) Gewirtz et al., 2014	181 women; 34 deployed mothers and 147 non-deployed mothers who had experience deployment of a partner/spouse.	Baseline measure part of the ADAPT evaluation	PCL; APQ-9; PLOC; BERS-2	Deployed mothers reported significantly greater distress; more PTSD and depression symptoms, and more difficulties in emotion regulation than non-deployed; deployed and non-deployed mothers did not differ in their reports of couple adjustment, parenting, or child behavior.
(8) Herzog et al., 2011	54 Army National Guard service members and their spouses/partners	Non-experimental observational single cohort design that included a one-time survey	PCL-M; STS; HITS; RAFFT; CBCL	Parental PTSD symptoms associated with internalizing – but not externalizing – problems in children in this study. Spouse secondary PTSD symptoms mediated between soldier PTSD symptoms and child secondary traumatic stress symptoms.
(9) Khaylis et al., 2011	36 Army National Guard Soldiers with children who had been previously deployed to OEF or OIF	Cross-sectional	PC-PTSD; Parenting concern and parenting stress assessed by parents’ 1 item self-rating	Parents reported being concerned about their child-rearing practices and felt that parenting was more stressful after deployment.
(10) Lester et al., 2010	272 children aged 6–12 from 171 United States Army and Marine families	Cross-sectional	Child: CBCL, CDI, MASC Parent: BSI, PDS, PCL-M	Active duty parent PTSD symptoms predicted child depression, as well as CBCL internalizing and externalizing behaviors; greater parent symptoms related to greater child symptoms.
(11) Lester et al., 2016	150 active duty or reserve component service members with at least one child under the age of 10; 301 primary caregiving parents and 150 primary military parents who identified a focal child	Single stratified sample from the active duty family and reserve duty family database; Community norms comparison for child outcomes.	Child: ASQ-SE, PAS, SDQ. Parent: PHQ8, PCL-M, AUDIT, Parental sensitivity, FAD, marital instability.	Parental depressive and posttraumatic stress symptoms associated with impairments in social emotional adjustment in young children, increased anxiety in early childhood, and adjustment problems in school-age children. Parental sensitivity associated with improved social and emotional outcomes across childhood.
(12) Mustillo et al., 2014	206 National Guard members fathers	Cross-sectional	Parenting difficulties Combat exposure; PC-PTSD; PHQ-9	Symptoms of PTSD are not associated with more parenting difficulties.
(13) Sayers et al., 2009	199 Veterans recruited via VA (referred for behavioral health evaluation)	Cross-sectional	PHQ-9; MINI; Family difficulties	Among partnered veterans with children, PTSD was associated with children acting afraid or not acting warm toward the veteran
(14) Sherman et al., 2016	19 veteran fathers (recruited via VA hospitals)	Mixed method study	Qualitative	Veterans reported parenting difficulties PTSD symptom clusters, including avoidance, alterations in arousal and reactivity, and negative alterations of cognitions and mood.
(15) Sherman et al., 2015	19 veteran fathers (recruited via VA hospitals)	Qualitative and quantitative, sequential, mixed method study	10 interviews PCL-C; AUDIT-C; PHQ-9; DAR-5	Veterans indicated strong desire to communicate with children about PTSD but also discussed barriers to doing so.
(16) Sullivan et al., 2016	513 Veteran parents with at least a child	Cross-sectional	PCL-C; PHQ-15; Child functioning measured using a list of five challenges often experienced by children in military/veteran families.	Veterans with higher PTSD symptoms more likely to report concerns about adverse child functioning; female veterans were more likely to endorse adverse child functioning compared with male veterans.
(17) Tomassetti-Long et al., 2015	104 active duty parents with children under 13	Cross-sectional	DRS-15R;PSI-SF;PCL-M	Symptoms of PTSD accounted for parenting stress; dysphoria a unique predictor of parenting stress.
(18) Vaughn-Coaxum et al., 2015	318 single (*n* = 74) and partnered (*n* = 244) veteran parents with at least 1 dependent child	Random sample; (drawn from larger project)	DRRI-2; PCL-M; BDI-PC; BAI	Single parents reported significantly higher PTSD symptoms than partnered parents.
(19) Waliski et al., 2013	172 OEF/OIF National Guard veteran parents with at least one deployment to Iraq or Afghanistan	Baseline survey results from an evaluation of an ACT-based educational workshop	Parental measures for anxiety, depression, and PTSD combined to produce a single variable; Children’s problems identified by asking veterans to report whether their children had any adjustment problems.	Parental mental health symptoms associated with 171% increase in likelihood of service member reporting a child with an emotional, behavioral, or adjustment problem.
(20) Yablonsky et al., 2016	111 active duty Navy fathers with young children	Cross-sectional	PSI; PC-PTSD; PHQ-8	Symptoms of depression mediate association between deployment factors (exposure to combat, perceived threat) and increased parenting stress after deployment.

*Abbreviations of measures used in the reviewed studies (see original source for citations): AAQ-II, Acceptance and Action Questionnaire-II; APQ, Alabama Parenting Questionnaire; APQ-9, Alabama Parenting Questionnaire 9 Item; ASQ-SE, Ages and Stages Questionnaire: Social-Emotional; AUDIT, Alcohol Use Disorders Identification Test; BAI, Beck Anxiety Inventory; BDI-2, Beck Depression Inventory-2; BDI-PC, Beck Depression Inventory-Primary Care; BERS-2, Behavioral and Emotional Rating Scale, Second Edition; BSI, Brief Symptom Inventory; CBCL, Child Behavior Checklist; CDI, Children’s Depression Inventory; CSI, Couples Satisfaction Index; DAR-5, Dimensions of Anger Scale; DAS, Dyadic Adjustment Scale; DRRI-2, Deployment Risk and Resilience Inventory-2; DRS-15R, Dispositional Resilience Scale; FAD, Family Assessment Device; HITS, The Hurt-Insult-Threaten-Scream; MASC, Multi-dimensional Anxiety Scale for Children; MINI, Mini-International Neuropsychiatric Interview; PAI, Parenting Alliance Inventory; PAS, Spence Preschool Anxiety Scale; PCL, PTSD Checklist; PCL-C, PTSD checklist Civilian Version; PCL-M, PTSD checklist Military Version; PC-PTSD, Primary Care-PTSD Screen; PDS, Posttraumatic Diagnostic Scale; PHQ, Patient Health Questionnaire (15 items, 8 items, 9 items versions); PLOC, Parental Locus of Control Scale; PSI, Parenting Stress Index; PSI-SF, Parenting Stress Index – Short Form; PSOC, Parenting Sense of Competence; PSS, Parental Stress Scale; RAFFT, The Relax-Alone-Friends-Family-Trouble; SDQ, Strengths and Difficulties Questionnaire; STS, Secondary Trauma Scale.*

## Advancing Cognitive-Behavioral Interpersonal Theory to Parent-Child Functioning

**FIGURE 1:**
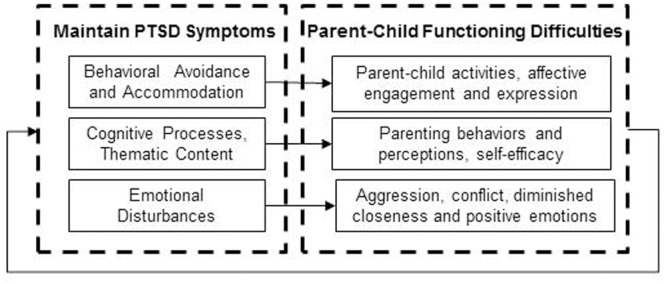
The cognitive-behavioral interpersonal theory of PTSD applied to parent-child difficulties.

The cognitive-behavioral interpersonal theory of PTSD (C-BIT) emphasizes the roles of three processes that both maintain PTSD symptoms and negatively impact intimate relationship functioning: (1) behavioral avoidance and accommodation, (2) cognitive processes and thematic content, (3) emotional disturbances (Dekel and Monson, 2010; Monson et al., 2010). We are not aware of prior extensions of this theory to parent-child functioning, however, we argue that the processes implicated in the association between PTSD and intimate relationship functioning are applicable to parent-child functioning (see **Figure 1**).

Within this model, behavioral avoidance and accommodation refer to the process through which PTSD avoidance symptoms are negatively reinforcing and thereby maintain trauma-related distress (e.g., Monson et al., 2012). The parent-child relationship may be adversely impacted if behavioral avoidance symptoms interfere with participating in parent-child activities or emotional numbing symptoms interfere with affective engagement and expression (e.g., Sherman et al., 2016). Supporting this view are several studies from Vietnam era samples implicating the avoidance symptoms of PTSD as being particularly deleterious to parenting (Ruscio et al., 2002; Samper et al., 2004). In turn, the family system, including children, may accommodate avoidance symptoms by facilitating avoidance behaviors, thereby further reinforcing this process and maintaining PTSD symptoms. For example, family members may modify their own activities so that one member may not encounter reminders that cause trauma-related distress (such as not attending a school function because a parent finds crowds distressing). Accommodation behaviors have only recently begun to be researched in intimate relationships (e.g., Fredman et al., 2014); further work is needed to examine accommodation in parent-child relationships.

Within the C-BIT model, cognitive processes and thematic content refer to rigid and maladaptive schemas about the world and past experiences and disruptions to core themes such as power, trust, control, and intimacy (Resick et al., 2014). Cognitive processes associated with PTSD such as attention bias toward threat and amplified attention to safety may influence parental perceptions of their child’s behaviors as more negative and enhance concerns about safety, and this is likely to influence parenting behaviors. Cognitive biases may also influence parental self-efficacy. For example, some veterans describe negative evaluations of themselves as parents, feelings of unworthiness as a parent, and alienation or detachment from their children (Sherman et al., 2016).

Emotional disturbances such as blunted positive emotions and increased anger, shame, guilt, and sadness are common to PTSD are thought to further impair family processes through disruptions to closeness, positive emotional experiences and emotional expression (Monson et al., 2012). For example, recent work has linked the dysphoria symptom cluster of PTSD to increased parenting stress in combat-exposed veterans (Tomassetti-Long et al., 2015). In their recent qualitative work, Sherman et al. (2016) noted that many veterans with PTSD spoke about the negative influence of emotional disturbances on their parenting. The influence of anger and aggression on parenting was another theme that emerged in Sherman’s study (2016), a finding that has been well-validated in two quantitative studies drawn from the United States National Comorbidity study and its replication. Specifically, parents with PTSD were more likely to report moderate and severe physical aggression with their children (Leen-Feldner et al., 2011), and the emotional numbing symptoms of PTSD were predictive of parent-child aggression (Lauterbach et al., 2007).

## Influence of Parent-Child Functioning on PTSD

At its core, the C-BIT model is bidirectional, suggesting that while PTSD influences family functioning, family functioning also influences PTSD. Several recent studies of veterans seeking healthcare at United States Veterans Affairs facilities provide initial support for an influence of parent-child functioning on PTSD. A study of the records of over 100,000 veterans found those with dependent children were about 40% more likely to have a diagnosis of PTSD during their first year of VA use, compared to counterparts without dependent children (Janke-Stedronsky et al., 2016). In another study, having minor children living in the home was related to increased PTSD symptom severity (Jobe-Shields et al., 2015). In a sample of veterans who had recently deployed, Vaughn-Coaxum et al. (2015) found that compared to partnered parents, single parents reported significantly greater post-deployment symptoms of PTSD even when controlling for combat exposure. Collectively this work suggests parenting status may enhance risk for PTSD symptoms after combat trauma exposure, particularly in single parents. One explanation for the association between parenting status and increased PTSD symptoms may be related to concerns about the well-being of family members while in the warzone, and how such concern may amplify perceptions of threat (Vogt et al., 2011). In addition, higher levels of parent-child functioning problems are likely to amplify PTSD symptoms, particularly during times of stress.

## Influence of PTSD on Parenting Outcomes Including Parenting Behaviors

Across samples of United States service members and veterans with recent deployments to the wars in Iraq and Afghanistan, symptoms of PTSD as well as depression have been implicated as predictors of parent-reported difficulties on a variety of indicators of parent-child functioning. For example, in samples comprised primarily of male National Guard/Reserve parents with recent deployments, PTSD symptoms were associated with concerns about child-rearing (Khaylis et al., 2011). In two other studies symptoms of depression but not PTSD were associated with perceived parenting difficulties (Mustillo et al., 2014) and increased parenting stress (Blow et al., 2013).

In a study of United States National Guard/Reserve fathers enrolled in a clinical trial to improve parenting after deployment, fathers, partners, and children were observed during a 5-min video interaction of a structured task (Brockman et al., 2016). Interactions were coded for the occurrence of positive engagement, withdrawal, avoidance and reactivity-coercion behaviors. At the univariate level, PTSD symptoms were associated with increased distress avoidance (fear, wariness, ignoring or low empathy in response to aversive behavior or affective distress of the child) during the observed interaction with children (Brockman et al., 2016). Mediational analyses indicated experiential avoidance (unwillingness or inability to remain in contact with negative thoughts, feelings or sensations) appeared to diminish the association between PTSD and observed parent-child interactions patterns and was associated with less positive engagement with children.

A study of 34 National Guard/Reserve mothers who had previously deployed (data drawn from the same clinical trial as above) reported higher PTSD symptoms and depression compared to 147 non-deployed mothers whose partners were deployed, however, there was no difference between the two groups on measures of parenting behaviors, parenting efficacy, or parent reported child functioning (Gewirtz et al., 2014). In another study examining United States veteran mothers randomly sampled from one geographic region, results indicated no association between PTSD symptoms or alcohol misuse with parenting satisfaction or confidence (Creech et al., 2016). Instead, parenting satisfaction was predicted by post-deployment stress exposure (number of life stressors after deployment such robbery or job loss). Results from both of these studies must be interpreted cautiously, however, due a small sample size of mothers who had previously deployed.

In a sample of active duty United States Army fathers (drawn from a study of the effectiveness of a marriage education workshop), baseline PTSD symptoms were associated with decreased parenting alliance, a measure of cooperation and communication between parents (Allen et al., 2010). In a random sample comprised primarily of active duty service members, Tomassetti-Long et al. (2015) reported that the dysphoria component of PTSD was a unique predictor of parental stress in recently deployed fathers, though it should be noted that parenting stress in the sample overall was very low. This study also found the personality variable hardiness to be protective against parenting and posttraumatic stress. In a sample of active duty United States Navy fathers, symptoms of depression but not PTSD appeared to mediate an association between several deployment factors (exposure to combat, perceived threat) and increased parenting stress after deployment (Yablonsky et al., 2016).

In a sample of United States Veteran parents referred for evaluation, PTSD symptoms and the psychomotor symptoms of depression were associated with increased likelihood of reporting “children acting afraid or not being warm” (Sayers et al., 2009). Within another treatment seeking group of Veterans, Sherman and colleagues examined veteran perspectives on the influence of PTSD on parenting. Interviews focused on communication with children about PTSD among a sample of 19 veteran parents who were diagnosed with PTSD. Veterans commented on barriers to talking with children about PTSD as well as motivations to do so, however, many also indicated such communication was challenging (Sherman et al., 2015). Results from a second study described themes related to the influence of specific PTSD symptom clusters on aspects of parenting (Sherman et al., 2016). Avoidance symptoms were described as interfering with participating in children’s activities and alterations in cognitions and mood were described as influencing self-worth as a parent, family engagement, and attachment. Importantly, some veterans described the influence of irritability and anger on their parenting behavior, with some describing incidents of aggression or aggressive urges toward children.

We were only able to find two studies in recent samples of veteran and military families that examined observed or self-reported parenting behaviors. Increases in PTSD symptoms 1 year after returning from a combat deployment to Iraq were associated with self-report of poorer parenting behaviors (less positive parenting, more inconsistent discipline, and less supervision) (Gewirtz et al., 2010). However, in a second study using observed parenting behaviors, there was no association between post-deployment PTSD symptoms with parenting behaviors (Davis et al., 2015). The authors suggested the difference in findings may be that observed parenting behaviors are only impaired at higher levels of PTSD symptoms and noted that a relatively small percentage of both samples exceeded cut-offs for probable PTSD.

## Influence of PTSD on Child Outcomes

Lester et al. (2010) examined behavioral and emotional problems of 272 children aged 6–12 from 171 United States Army and Marine families with a parent currently or recently deployed. Although children’s scores on depression and externalizing/internalizing (according to both parent and self-reports) were comparable to normative data; active duty parent PTSD symptoms predicted child depression, as well as internalizing and externalizing behaviors. Lester et al. (2016) also examined the relationship between parental PTSD and children’s outcomes in a national probability sample comprising 150 active duty or reserve component service members with at least one child under the age of 10. The findings highlighted a small but significant association between military parental PTSD severity and preschool child separation anxiety, as well as increased emotional and behavioral problems in school-aged children.

In another study exploring secondary traumatic stress symptoms in spouses and children of 54 National Guard families, Herzog et al. (2011) found a moderate relationship between PTSD symptoms in service members and secondary trauma symptoms in children. Children’s internalizing but not externalizing problems were symptomatic of secondary trauma stress in children, and were mediated by the spouse’s secondary trauma stress symptoms. However, only seven families were identified with high levels of military-related PTSD.

Several studies examined parent’s perceptions of children’s symptoms. Waliski et al. (2013) reported on a sample of 172 National Guard parents who were asked whether they had a child with any emotional, behavioral, or adjustment problems. Parents who reported greater mental health symptoms (including, but not specific to PTSD) and lower satisfaction with family and social relationships also reported having a child with problems. Overall, mental health symptoms resulted in a 171% increase in the likelihood of a service member reporting a child with an emotional, behavioral, or adjustment problem.

Similarly, Sullivan et al. (2016) surveyed a non-random sample of 513 veterans living in southern California), who reported having at least one child. Child functioning was measured using a list of five challenges often experienced by children in military/veteran families (i.e., concerns about behavior, academic performance, peer relationships, emotional problems, and physical problems). Although most veterans did not report significant symptoms, veterans who reported higher levels of PTSD symptomatology were also more likely to report negative perceptions about their child’s functioning. Veteran fathers were significantly less likely to report concerns about negative child functioning compared with veteran mothers.

Though collectively these studies suggest that parental PTSD symptoms may increase the likelihood of parents reporting adjustment problem in their children, it is important to note that in the absence of standardized measure or cross-informant reports, a parent’s perception alone may not be an accurate nor reliable indicator of the actual behavioral problems of the child. It is possible that such reporting could be an indication of cognitive bias inherent in PTSD, which may lead a family member with PTSD to perceive more negative behavior in their close family members, which will be consistent with C-BIT theory (Dekel and Monson, 2010).

## Conclusion

With regard to parenting outcomes, the studies reviewed indicated there is an association between parental PTSD symptoms in male military service members or veterans with self-reported parent-child functioning difficulties (Sayers et al., 2009; Allen et al., 2010; Khaylis et al., 2011). Consistent with the C-BIT model’s emphasis on the deleterious impacts of emotional disturbances on family functioning, new research indicates symptoms of depression may play an important role in parent-child functioning after deployment (Blow et al., 2013; Mustillo et al., 2014; Yablonsky et al., 2016). Several studies identified promising mechanisms through which PTSD symptoms may influence specific parenting difficulties – a considerable advance beyond what was previously available. For example, Brockman et al. (2016) found that while PTSD symptoms influenced parental distress avoidance, higher experiential avoidance was associated with less positive engagement with children. The work of Sherman and colleagues delineated several ways in which PTSD symptoms may directly influence parent-child functioning by implicating communication as a problem area and by linking specific symptoms of PTSD to areas of difficulty. With regard to other parenting behaviors, findings were somewhat conflicting, suggesting an association between PTSD with self-reported poorer parenting behaviors, but not with observed parenting behaviors (Gewirtz et al., 2010; Davis et al., 2015). More work is needed in this area. The two studies conducted in samples of women veterans and service members (Gewirtz et al., 2014; Creech et al., 2016) underscore the need for further work to understand the influence of military-related PTSD on parent-child functioning of mothers.

With respect to parental PTSD and child outcomes, there appears to be consensus that in active duty samples, parental PTSD symptoms have an effect on children’s internalizing and externalizing symptoms, including depression, social emotional adjustment in young children, increased anxiety in early childhood, and adjustment problems in school-age children. However, these studies do not provide any insight whether the child outcomes are impacted differently if the active duty parent was the mother or the father. When such distinction is made, in veteran samples (e.g., Gewirtz et al., 2014; Sullivan et al., 2016), the results appear contradictory and suggest that female veterans were more likely to endorse adverse child functioning compared with male veterans. Studies comprising National Guard samples suggest a relationship between parental PTSD and child internalizing but not externalizing problems. However, few studies utilized standardized measures of child outcomes.

## Directions for Future Research

The studies included in this review underscored several important considerations for future research. First, we were unable to find studies undertaken in non-United States populations. This may be a function of the databases used in the review or the requirement that studies were published in English; however, it is imperative that research begins to measure these processes in other countries. Second, as the number of women in the military continues to increase (at least in the United States) more work must oversample for women so that an understanding of the influence of military-related PTSD on mothers can be developed. Third, although work has been separately undertaken in United States reserve component service members as well as in active duty samples, less work is available that enables a comparison between the two. This may be important as the resources available to active duty are different than those available to reserve component service members and veterans. Finally, longitudinal and nationally representative studies that utilize standardized measures of PTSD and child outcomes will enable comparisons across studies and with community samples.

## Author Contributions

Both authors contributed to literature searches and writing of this paper. Both authors approved the final manuscript.

## Disclaimer

The contents of this manuscript are those of the authors and do notnecessarily represent the views of the Department of Veterans Affairs, or the United States Government.

## Conflict of Interest Statement

The authors declare that the research was conducted in the absence of any commercial or financial relationships that could be construed as a potential conflict of interest.
